# Natural SARS-CoV-2 infection in farmed minks *(Neovison vison)* causes lung pathology, systemic viral spread, and transmission risk, even in asymptomatic animals

**DOI:** 10.3389/fvets.2026.1752459

**Published:** 2026-03-24

**Authors:** Sandra Vreman, Giuseppe Giglia, Robert-Jan Molenaar, Renate Hakze-van der Honing, Eveline M. Delemarre, Stefan Nierkens, Katrin E. Wiese, Gianfilippo Agliani, Wim van der Poel, Frank van Kuppeveld, Berend Jan Bosch, Erwin de Bruin, Andrea Gröne, Naomi de Bruijn, Judith M.A. van den Brand

**Affiliations:** 1 Wageningen Bioveterinary Research, Wageningen University and Research, Lelystad, Netherlands; 2 Division of Pathology, Department of Biomolecular Health Sciences, Faculty of Veterinary Medicine, Utrecht University, Utrecht, Netherlands; 3 Department of Veterinary Medicine, University of Perugia, Perugia, Italy; 4 Department of Poultry Health, Royal GD, Deventer, Netherlands; 5 Center for Translational Immunology, University Medical Center Utrecht, Utrecht, Netherlands; 6 Section of Virology, Division of Infectious Diseases & Immunology, Department of Biomolecular Health Sciences, Faculty of Veterinary Medicine, Utrecht University, Utrecht, Netherlands

**Keywords:** clinical disease, commercial farm, lung pathology, mink, SARS-CoV-2

## Abstract

**Introduction:**

In 2020, the first disease outbreak of severe acute respiratory syndrome coronavirus 2 (SARS-CoV-2) in farmed minks *(Neovison vison)* was reported in the Netherlands, followed by outbreaks in other countries. The disease in minks is characterized by interstitial pneumonia and viral replication in both the upper and lower respiratory tracts, resulting in respiratory disease and, in some cases, death. A major concern, besides animal health problems, is that minks are a potential reservoir for SARS-CoV-2, with a zoonotic impact for human Coronavirus Disease-19 (COVID-19), underscoring the need for close monitoring of infections.

**Methods:**

To better understand the dynamics of viral spread and disease progression after natural infection, this study investigates the pathology, immunohistochemistry, virology, serology, proteomics, and the presence of Aleutian Disease Virus (ADV) in minks from an infected farm. A total of 45 minks were divided into four groups based on clinical health status and time of sampling: found dead before culling (FD, *n* = 15), clinically healthy during culling (NCSc, *n* = 10), clinical signs during culling (CSc, *n* = 10), and found dead during culling (FDc, *n* = 10).

**Results:**

Histopathological examination revealed that interstitial pneumonia was the most prominent SARS-CoV-2-related finding across all four groups, with more severe lesions seen in the FD and FDc groups. Histopathological changes were supported by viral antigen expression in the nose, trachea, and lungs, as well as in extra-respiratory tissues such as the intestine, spleen, and lymph nodes, in all groups. Highest viral RNA levels were found in nasal and throat swabs and nose and lung tissues. Lower levels were detected in the spleen, liver, and intestine, especially in the FDc animals. Serology confirmed the presence of SARS-CoV-2 specific antibodies in all groups, whereas immune-related proteomics on whole blood did not show a significant difference between the groups. Based on qPCR, over 57% of minks were co-infected with ADV.

**Discussion:**

Minks naturally infected with SARS-CoV-2 exhibit severe lung pathology and high viral loads across multiple organs. Additionally, severe lung lesions are also observed in animals without clinical signs, suggesting a potential zoonotic risk and viral spread in the absence of any clinical signs.

## Introduction

Severe acute respiratory syndrome coronavirus 2 (SARS-CoV-2) is the cause of Coronavirus Disease-19 (COVID-19) in humans. Starting from 2020, natural infection has also been reported in many other animal species (1). SARS-CoV-2 was first reported in farmed minks in the Netherlands (2, 3), soon followed by other countries (4, 5) in Europe (6, 7) and in the USA (8, 9), suggesting a marked susceptibility of this species to SARS-CoV-2 infection and disease (10, 11), which could be related to the presence of the virus binding angiotensin converting enzyme 2 (ACE2) receptor in the lower respiratory tract of minks (12). COVID-19 infection in mink mainly results in respiratory disease characterised by interstitial pneumonia and replication of the virus in both the upper and lower respiratory tract, potentially leading to clinical respiratory disease and death (2, 13). The macroscopic pattern of lesions in minks is characterized by mottled to diffusely dark-red and wet lungs, histologically signified by areas with classic features of diffuse alveolar damage (DAD), such as hyaline membranes, type II pneumocytes proliferation, and interstitial infiltration of mononuclear cells in acute stages (2, 3). Although similar clinical and pathologic findings were observed in all mink cases, lesion severity varied. Unfortunately, no data regarding the progression of the clinical disease and the lesion severity have been reported for naturally infected minks (14, 15). Besides animal health implications, the potential role of this species in the human-to-animal and animal-to-human zoonotic transmission (7, 15, 16) and its possible role as a reservoir (17), raised additional concern (15, 18).

SARS-CoV-2 associated disease in naturally infected minks shows clinical and pathological findings that are overlapping with what is described for the infection in humans. In both minks and humans, the acute phase of disease is characterized by DAD in the lung, with alveolar oedema, hyaline membranes, and vascular congestion (2, 9, 19, 20). The progression to the proliferative/organizational subacute phase of DAD leads to type II pneumocyte hyperplasia with eventual squamous metaplasia and fibroblastic proliferation in the chronic fibrotic stages of DAD, observed in both humans and minks. However, in humans, the fibrotic phase of DAD involves the entire lung, with more pronounced lesions than in minks. Furthermore, human lungs more often show vascular changes, including fibrinous thrombi and parenchymal infarcts, which are rare or absent in minks (2, 9, 19, 20).

It is expected that, in minks, like in humans, concomitant infections may affect the clinical course of the SARS-CoV-2 infection. A potentially significant co-infection most likely preceding the SARS-CoV-2 infection, could be the immunosuppressive Aleutian disease (AD), caused by the Carnivore amdoparvovirus-1, also known as Aleutian Disease virus (ADV), which was endemic in the Netherlands at the time (21), leading farmers to breed for tolerance (22, 23). In adult mink, AD is a chronic and progressive infection that elicits an abnormal immune response, causing immune-complex-mediated damage and plasma cell-rich infiltrates, accompanied by lymphocytes and histiocytes, in multiple organs, including the kidneys, liver, brain, and lungs. This has led the disease to be sometimes referred to as mink plasmacytosis. In young minks, AD can lead to acute alveolar cell infections, with interstitial pneumonia, type II pneumocyte hyperplasia, and hyaline membrane formation (24, 25). Co-infections in SARS-CoV-2 infected humans can be prevalent in up to 50% of deceased patients (26). Common pathogens involved in co-infections include various bacteria, viruses (mainly influenza A), and fungi (such as *Aspergillus* spp.) (27). These co-infections may impact the course of the disease and increase the risk of mortality. Interestingly, the presence of ADV in SARS-CoV-2 infected minks mimics a similar situation in humans, where a dysregulated immune system by a co-infection can significantly influence the course of COVID-19 (27).

Due to the similarities of the acute stages of the disease between farmed minks and humans, minks could not only represent a relevant model to evaluate the pathogenesis of natural SARS-CoV-2 infection, but also be a valuable model to monitor newly circulating viral strains for their evolution, transmission, and clinical impact in a large group of hosts living in proximity to each other. To this end, the current study reports on SARS-CoV-2 related parameters in different clinical categories, possibly reflecting various stages of disease, on a culled mink farm. This includes SARS-CoV-2 related histopathology, immunohistochemistry, virology, and serology of clinical healthy, ill, and deceased minks on a SARS-CoV-2 infected farm, with or without a chronic ADV co-infection.

## Materials and methods

### Farm and animals

All sampled minks (*Neovison vison*) originated from the same Dutch farm, housing 1,800 breeding females and their 6,200 kits in standard sheds. This entire mink population was ‘wild type’ with a brown pelt colour. The farm had been positive for ADV for many years and was breeding for ADV resistance (22, 23). To this extent, the farmer relied on the iodine agglutination test to remove animals with high amounts of serum gamma globulin from the breeding stock before the start of the breeding season (28, 29). The farmer stated that no signs of clinical disease due to ADV had been recognized in the past few years and there was only incidental ADV related mortality (not routinely diagnosed or recorded) showing gross lesions consistent with chronic ADV such as renal and splenic swelling. Besides ADV there were no recent health problems on the farm, aside from incidental bacterial disease in individual mink. Before the outbreak of SARS-CoV-2, the farm had been sampled weekly as part of the mandatory Dutch ‘Early Warning’ (EW) programme for SARS-CoV-2 and always tested negative. According to this programme, all Dutch mink farms must submit the carcasses of recently deceased minks weekly for testing for SARS-CoV-2 using PCR on pooled throat swabs. On June 3, 2020, the farm participated in a serological survey. On this occasion, animals were inspected by a veterinarian for clinical signs of COVID, and blood samples were collected from 60 minks across the farm. These minks tested negative for SARS-CoV-2 antibodies using an in-house ELISA test.

### SARS-CoV-2 outbreak, clinical signs and study set-up

**Figure 1:**
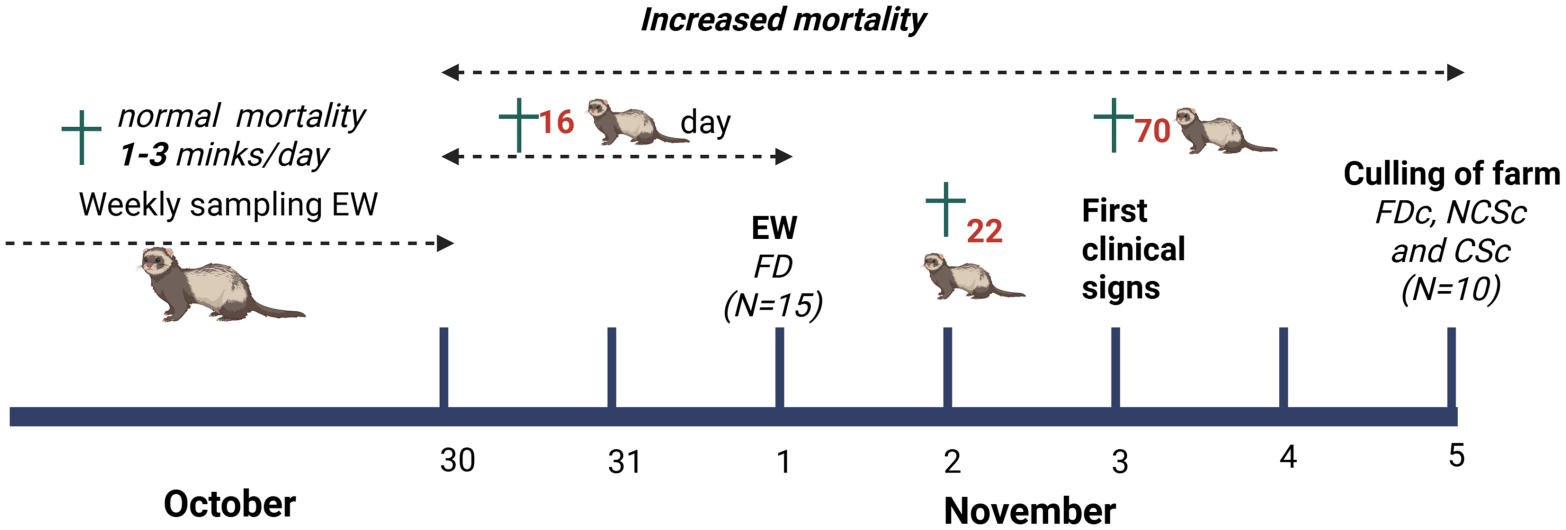
Timeline SARS-CoV-2 outbreak and sampling. Before the outbreak, carcasses of recently dead minks were submitted weekly for the early warning program (EW). From October 30th, mortality increased, and the farm was culled on November 5th. FD, found dead EW; FDc, found death culling; NCSc, no clinical signs culling; CSc, clinical signs culling. Created with BioRender.

On both the 30th of October and 1st of November 2020, there was a sudden spike in mortality: 16 minks were found dead per day, while normal mortality varied between 1 and 3 minks per day (Figure 1). On the 2nd and 3rd of November, mortality further increased to 22 and 70 animals, respectively, prompting the farmer to inform the authorities of a suspected SARS-CoV-2 outbreak. After this day, no further records were kept of mortalities. On November 3rd, the farmer noticed the first clinically sick animals. These minks had mucoid nasal discharge, epistaxis, laboured breathing, or a combination of these signs. On the previous days, no such signs were seen, and mortality occurred in apparently healthy animals that stopped eating for a day and were then found dead subsequently. Fifteen dead minks from the 1st of November were sent in for the EW programme, and three out of four pools of throat swabs of these animals tested positive for SARS-CoV-2 in PCR. These fifteen carcasses with minimal autolysis were subjected to additional individual sampling for this study (group 1, found dead (FD) *n* = 15). On the 5th of November, the whole mink population on the farm was culled. All minks were humanely killed using carbon monoxide and freshly euthanised minks were collected for additional sampling: ten randomly selected minks without signs of disease (group 2, NCSc, *n* = 10); ten randomly selected minks with clinical signs of respiratory disease and/or mucoid nasal discharge (group 3, CSc, *n* = 10) and ten randomly chosen carcasses of minks that were found dead on that day from different farm sheds were also collected (group 4, FDc, *n* = 10) for testing. No selection by age or sex was possible, since mothers and pups were housed together. Animals were randomly collected from cages across the farm (see overview Table 1 and Supplementary File 1 for details on minks).

**Table 1:** Group overview.

Group	Name	Mink nr	Group details
1 (*n* = 15)	FD	#1–15	Found dead on 1st November
2 (*n* = 10)	NCSc	#16–25	No clinical signs, culled on 5th November
3 (*n* = 10)	CSc	#26–35	Clinical signs, culled on 5th November
4 (*n* = 10)	FDc	#36–45	Found dead on 5th November during culling

FD, found dead; NCSc, no clinical signs culling; CSc, clinical signs culling; FDc, found dead culling.

### Pathology and immunohistochemistry

For all 45 animals, a comprehensive post-mortem investigation was conducted, including evaluation of macroscopic lesions. The following tissues were collected in neutral buffered 10% formalin: conchae, brain, trachea, trachea-bronchial lymph node, lung, liver, spleen, kidney, jejunum, colon, skin, and eye. After fixation in 10% buffered formalin, all samples were trimmed, routinely processed, and embedded in paraffin. For histology, 3-micron thick sections were cut from formalin-fixed and paraffin-embedded tissues (FFPE) and routinely stained with haematoxylin and eosin (H&E). For all 45 animals, histological examination of all tissues was performed using a light microscope (Olympus BX50). In the lung, microscopic lesions were staged (mild, moderate, and severe) based on the extent of the lesion (mild < 25%, moderate 26–50%, severe > 51%), subdivided into epithelial, vascular, fibrotic, and other reactions of the different stages of DAD (exudative, proliferative, fibrosing phases) described in animals (2, 9, 30) and in humans (31–34). Lesion assessment was performed according to the epithelial, vascular, fibrotic, and other lesion characteristics of the DAD phases, as reported in the literature (26), to evaluate the different clinical categories of the examined animals and assess possible evolution of the pulmonary lesions compared with the human disease.

Immunohistochemistry (IHC) for SARS-CoV-2 was performed on 3-micron thick sections of the respiratory tracts of all cases. For the intestinal tract, all cases with available tissue in FFPE blocks were assessed (*n* = 36/45). At the same time, a subset of animals was selected from the different groups for other organs (17 cases for the brain, and 14 cases for the liver, spleen, heart, kidney, tonsil, lymph node, skin, and eye). Tissue sections were deparaffinized in xylene followed by rehydration (decreasing alcohol series of 90, 80, 70, and 50% concentration). Antigen retrieval was performed in citrate buffer (pH 6) at 97 °C. Endogenous peroxidase blocking was performed with Dako Peroxidase Blocking Solution for 5 min, after PBS/Tween 20/BSA immersion. An additional blocking step was performed using goat serum for 15 min. Primary antibody incubation, (human SARS coronavirus nucleoprotein antibody 40143-T62, Sino Biological Europe GmbH, Eschborn, Germany) at a 1:10,000 dilution was performed for 60 min. As a secondary antibody, commercial Brightvision Goat anti-mouse/rabbit HRP was used for 30 min. Finally, visualization of the antigen was performed with AEC for 30 min. Slides were finally counterstained with Haematoxylin and mounted with an aqueous medium (Aquatex, VWR, Leuven, Belgium). The presence of SARS-CoV-2 antigen on IHC was scored with a scoring system defined according to the proposed literature criteria (35). The scoring system was based on the number of positive cells counted in 10 randomly selected high-power fields (HPFs; 2.37 mm^2^ tissue area) (each field diameter of 0.55 mm, HPF area of 0.237 mm^2^), and graded as follows: 0 when no signal was detected; grade 1 for 1–100 labelled cell(s); grade 2 for 101–200 labelled cells; grade 3 for ≥ 201 labelled cells.

### SARS-CoV-2 and Aleutian disease virus detection

**Figure 2:**
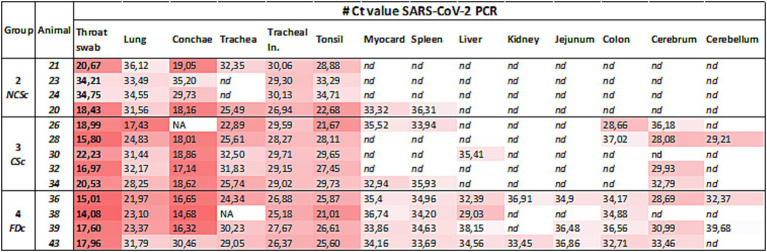
SARS-CoV-2 viral RNA measured by RT-qPCR. From each culled mink group, 4–5 animals with throat swabs showing high and low Ct values were selected. For these minks, additional organs were investigated for viral RNA; red graded color scale, low Ct values are more red; not analyzed (NA); not detected (nd).

For all 45 minks, throat swabs, nose swabs, rectal swabs, and available lung tissue, nasal conchae, and brain (cerebrum) (Supplementary File 1) were analysed for viral RNA using an E gene SARS-CoV-2 PCR assay as described previously (3, 36). RNA was extracted from the swabs and the 10% organ homogenate using the Directzol RNA kit (Zymo Research) as previously described (3). The real-time RT-PCR was performed in a 20 μL reaction containing 10 μL of 2x TaqMan Fast virus 1-step PCR mix, 400 nM of each primer, and 200 nM of the probe. Thermal cycling was performed at 55 °C for 10 min for reverse transcription, followed by 95 °C for 20 s, and then 45 cycles of 95 °C for 15 s, 58 °C for 30 s on a LightCycler 480 (Roche). Additionally, four representative animals (2 with high Ct value and 2 with low Ct value throat swab) were selected from groups NCSc (animals #20, 21, 23 and 24), CSc (animals #28, 30, 32 and 34) and FDc (animals #36, 38, 39 and 43), to investigate systemic viral distribution in heart, spleen, liver, kidney, jejunum, colon, and brain (cerebellum) (Figure 2). For the CSc group, animal #26 was additionally investigated while no animals from group FD could be selected, as this group had been sampled at an earlier time point before the farm was culled.

The farmer considered ADV to be endemic on this mink farm. To investigate the ADV status of the individual animals, a qPCR was performed on all collected spleen samples. DNA was extracted from the 10% spleen homogenate with the QIAmp MinElute Virus Spin Kit. The Real-time PCR was performed in a 20 μL reaction containing 10 μL of 2x Taqman Fast virus 1-step PCR mix. 400 nM Forward primer Fw-3493-ADV AATGGATAATACCAGCAGGGTTAC and 400 nM reverse primer Rv1-3716-ADV GGTTGCTTTGCTGTATGTTGC and 200 nM probe P1-3519-ADV 6-FAM GGTAGTTACTTTGCTGGAGGACCA BHQ-1 was added to the mix together with 5 μL of template DNA. Thermal cycling was performed at 95 °C for 20 s, followed by 45 cycles of 95 °C for 10 s and 60 °C for 30 s, on a LightCycler 480 (Roche).

### Virus sequencing

From all groups, 3–5 animals were randomly selected to determine SARS-CoV-2 sequences of the throat swabs by next-generation sequencing as described previously (3). All generated sequences were deposited in the GISAID database: EPI_ISL_19698991 up to EPI_ISL_19698999. Selected animals: FD #8–10–12-15; NCSc #18–20-21; CSc #26–27–28-31-33-35; FDCc #36–39–41-42.

### Serology

Serum was derived from (clotted) blood samples, collected during necropsy from all minks (*n* = 45). Serum samples were analysed using the Wantai SARS-CoV-2 Ab ELISA (Wantai Biological Pharmacy, Beijing, China) (37), according to the manufacturer’s instructions.

The ability of serum samples to neutralize the virus was assessed using a SARS-CoV-2 pseudoviral neutralization assay, as previously described (38). Briefly, for production of SARS-CoV-2 spike (S) pseudotyped VSV, HEK-293 T cells (70–80% confluency) were transfected with pCAGGS expression vectors encoding the SARS-CoV-2 spike protein, which included an 18-residue truncation at the C-terminal cytoplasmic tail to enhance surface expression. At 48 h post-transfection, the cells were infected with VSV G-pseudo-typed VSVΔG containing the firefly luciferase (*Photinus pyralis*) reporter gene. The supernatant was collected 24 h later, filtered through a 0.45-μm membrane, and used to generate pseudo-typed VSV. Virus titration was performed by infecting VeroE6 cells. For the pseudovirus neutralization assay, sera were serially diluted threefold (starting dilution fold of 50) and pre-incubated with an equal volume of virus for 1 h at room temperature. Then, the serum was added to VeroE6 cells for infection. After 20 h of incubation at 37 °C, the cells were washed once with PBS and lysed using Passive Lysis Buffer (Promega). Firefly luciferase activity was measured with a Berthold Centro LB 960 plate luminometer using d-luciferin (Promega) as the substrate. The percentage of neutralization was calculated by comparing the luciferase reduction in the presence of serum to the luciferase signal obtained in the absence of serum (38). The sample neutralization titers were determined by the reciprocal of the highest dilution that resulted in >90% reduction of luciferase activity (VNT90).

### Immune-related proteomics

Whole blood samples (frozen −80 °C) were used for proteomic measurements of 12 animals in total: animals #16, 17, 18, 19, and 20 (NCSc, *n* = 5); #27, 28, 29, and 30 (CSc, *n* = 4), and #36, 37, and 39 (FDCc, *n* = 3). The animals selected from each group were the most representative with respect to pathology and virology. The blood samples were analysed using proximity extension assay (PEA) technology based Proseek Multiplex panels (Olink Proteomics, Immuno-oncology panel including 92 immune-related proteins), performed by the Olink provider in UMC Utrecht, the Netherlands. This Olink method was not validated for whole blood samples derived from minks. In short, PEA technology utilizes antibody sets linked to matching DNA-oligonucleotides specific to each protein of interest. These oligonucleotides hybridize when brought into proximity after binding the protein and are extended by DNA polymerase, thereby forming PCR targets. These PCR targets are quantified by real-time PCR. The quantification cycle values were converted into normalized protein expression (NPX) units. The NPX values were expressed on a log2 scale, in which a one-unit increase in NPX values represents a doubling of the measured protein concentration. Quality controls were measured on every plate using Olink’s standard quality control protocol. As the samples were not measured on the same plate, bridging controls (*N* = 12) were included to correct for batch effects between the measurements using BAMBOO, an in-house developed method (39).

### Statistics

Statistical analysis of the pathology and immunohistochemistry assessments was conducted in RStudio (version 2024.12.0). The objective was to evaluate differences in alveolar damage severity, pneumonia severity grade, and SARS-CoV-2 IHC-positive cell counts among the four groups. For pathology, to assess the association between the alveolar damage severity grade, pneumonia severity grade, and the groups, a Chi-square test was conducted. For the SARS-CoV-2 IHC-positive cell counts, variables analysed included lung-positive cell count, nasal-positive cell count, and tracheal-positive cell count. To determine whether the data followed a normal distribution, the Shapiro–Wilk test was applied. To compare IHC cell counts across the four groups, the Kruskal-Wallis test was used. In cases of statistical significance, *post hoc* comparisons were performed using Dunn’s test with the Benjamini-Hochberg correction. Associations between viral load (SARS-CoV-2 Ct values) and ordinal outcomes (virus antigen in IHC and histopathology scores) were assessed using Spearman’s rank correlation coefficient (*ρ*). To control for multiple testing, *p*-values were adjusted using the Benjamini–Hochberg false discovery rate (FDR) procedure. An FDR-adjusted p-value < 0.05 was considered statistically significant. The association between lung viral load and lung antigen detection was evaluated using an ordinal logistic regression model with lung SARS-CoV-2 IHC virus antigen score as the outcome and SARS-CoV-2 lung Ct value as the predictor. Additional multivariable ordinal logistic regression models were constructed to evaluate whether lung viral load (Ct) and SARS-CoV-2 lung antigen detection in IHC were independently associated with lung pathology outcomes, including pneumonia severity score and diffuse alveolar damage score. Models were fitted using a logit link function under the proportional odds assumption. Regression coefficients were exponentiated to obtain odds ratios (ORs) with corresponding 95% confidence intervals (CI). Statistical significance was assessed using Wald tests, with two-sided *p*-values < 0.05 considered statistically significant. GraphPad Prism 8.1.1 one-way ANOVA post-hoc Kruskal-Wallis was used for ELISA, VNT90, and qPCR. The significance level was set at *p* < 0.05 for all the analyses.

## Results

### Pathology

Macroscopic lesions were most frequently seen in the respiratory tract and associated lymph nodes. In the upper respiratory tract, mucopurulent exudate was observed in all groups. The lungs showed a diffuse or patchy dark-red discoloration and/or oedema (i.e., wet lung cut-surface and intraluminal fluid in bronchi/trachea), and the associated lymph nodes were severely enlarged (Supplementary File 2). Other macroscopic lesions were seen in the heart, liver, and spleen. In the heart, left ventricular dilation was observed in 2 cases. The liver and spleen showed diffuse enlargement (hepato- and splenomegaly) in 7 and 11 cases, respectively, with an equal distribution across all groups.

**Figure 3:**
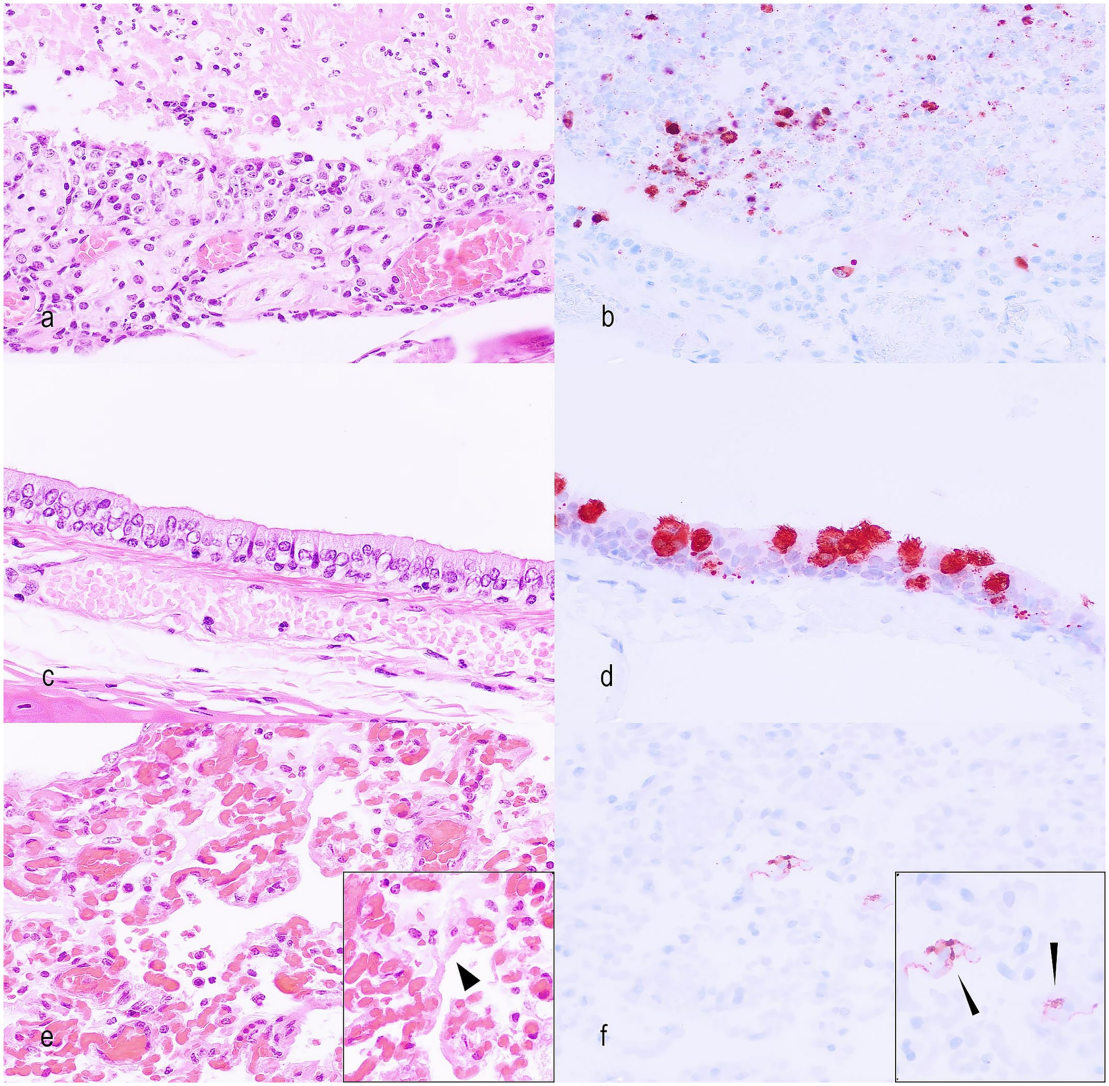
Representative lesions in the upper respiratory tract and lungs of SARS-CoV-2-infected minks. **(a)** Nasal cavity showing a severe mucopurulent rhinitis. Hematoxylin and eosin (#18; NCSc; HE; x400). **(b)** Nasal cavity showing a severe SARS-CoV-2 antigen expression in epithelial cells and luminal exudate (#18; NCSc; Virus antigen IHC; x400). **(c)** Trachea with minimal lesions represented by multifocal loss of cilia (#33; CSc; HE; x400). **(d)** Tracheal epithelium showing SARS-CoV-2 antigen in numerous epithelial cells (#33; CSc; Virus antigen IHC; x400). **(e)** Lung with diffuse alveolar damage represented by pneumocyte loss, fibrin, and hyaline membranes (short arrowhead in inset) (#38; FDc; HE; x400). **(f)** Lung showing SARS-CoV-2 antigen in type I pneumocytes (long arrowheads in inset) (#38; FDc; Virus antigen IHC; x400).

Histology of the upper respiratory tract (Figure 3) showed a muco-purulent rhinitis and a lymphocytic or neutrophilic tracheitis (Table 2). The rhinitis was frequently of higher severity (moderate to severe) in CSc (*n* = 9/10; 90%), FD (*n* = 10/15; 66%) and FDc animals (8/9; 89%—one section was not assessable due to severe autolysis), compared to NCSc, which showed more commonly absent to mild lesions (*n* = 8/10; 80%, Supplementary File 3). The lung histopathology showed acute and exudative stages of DAD in all investigated groups (*n* = 44/44; 100%, Supplementary File 3—one FDc case was not assessable due to severe autolysis), mainly characterised by hyaline membranes, sloughed-off pneumocytes (Figure 3), and interstitial and alveolar oedema. The pattern of these lung changes was also present in animals without clinical signs (NCSc). A higher proportion of animals in the CSc group exhibited severe lung changes (*n* = 9/10; 90%) compared to the FD (*n* = 9/15; 60%) and FDc groups (*n* = 6/9; 67%) (Supplementary File 4, Supplementary Figures 1, 2). Hyaline membranes were more frequent in FD and FDc than in NCSc and CSc, whereas syncytia formation showed similar frequencies in CSc and NCSc, and a lower frequency in FD and FDc. Reactive atypia (*n* = 29/44, 65%) and hyperplasia of type II pneumocytes (*n* = 2/44, 5%) were also observed, the first one more frequent in FDc (9/9; 100%) than in all other groups. None of the examined animals showed lesions of the fibrosing/chronic stage (*n* = 0/44; 0%). There was no significant association between alveolar damage and pneumonia severity grades across groups (*p* > 0.05). Additionally, some animals in each group, more frequently in CSc and FDc groups, presented a plasma-cell rich and histiocytic inflammation mainly in the brain (*n* = 12), liver (*n* = 9), eye (*n* = 5), heart (*n* = 2), kidney (*n* = 4), which are most likely not related to the SARS-CoV-2 infection, but to chronic ADV infection. Other organs investigated (spleen, skin, tonsils, and bronchial lymph nodes) showed no significant changes (Supplementary File 1 and Supplementary File 5).

**Table 2:** Summary of pulmonary histological patterns identified in SARS-CoV-2 naturally infected minks.

Pattern of lesions	Elementary lesions	Group 1 FD *N* = 15 (%)	Group 2 NCSc *N* = 10 (%)	Group 3 CSc *N* = 10 (%)	Group 4 FDc *N* = 9(%)*
Epithelial	Pneumocyte injury (Sloughing/detachment)	15/15 (100)	10/10 (100)	10/10 (100)	9/9 (100)
	Reactive atypical pneumocytes	5/15 (33)	7/10 (70)	8/10 (80)	9/9 (100)
	Syncytial cells	7/15 (47)	9/10 (90)	9/10 (90)	8/9 (89)
	Type II pneumocyte hyperplasia and hypertrophy	1/15 (7)	1/10 (10)	0/10 (0)	0/9 (0)
	Viral cytopathic changes (i.e., viral inclusions)	0/15 (0)	2/10 (20)	1/10 (10)	0/9 (0)
	Squamous metaplasia	0/15 (0)	0/10 (0)	0/10 (0)	9/9 (100)
Vascular	Capillary congestion	15/15 (100)	10/10 (100)	10/10 (100)	9/9 (100)
	Alveolar oedema	15/15 (100)	10/10 (100)	10/10 (100)	9/9 (100)
	Hyaline membranes	4/15 (27)	0/10 (0)	0/10 (0)	9/9 (100)
	Alveolar haemorrhages	9/15 (60)	10/10 (100)	10/10 (100)	7/9 (78)
	Microthrombi	0/15 (0)	0/10 (0)	0/10 (0)	0/9 (0)
Fibrotic	Myofibroblastic proliferation	0/15 (0)	0/10 (0)	0/10 (0)	0/9 (0)
	Alveolar granulation disuse	0/15 (0)	0/10 (0)	0/10 (0)	0/9 (0)
	Obliterating fibrosis	0/15 (0)	0/10 (0)	0/10 (0)	0/10 (0)
	Interstitial fibrosis	0/15 (0)	0/10 (0)	0/10 (0)	0/10 (0)
Other	Peribronchial/perivascular cuffing	15/15 (100)	9/10 (90)	9/10 (90)	9/9 (100)
	Megakaryocytes	12/15 (80)	10/10 (100)	10/10 (100)	9/9 (100)

FD(c), found dead before and during culling (c); NCSc, No clinical signs culling; CSc, Clinical signs culling; *one autolytic case.

IHC showed viral antigen expression in the upper and lower respiratory tracts, primarily in ciliated epithelial cells lining the mucosa of the nasal cavity, trachea, and lungs (Figure 3 and Supplementary File 6). Additionally, type I and type II pneumocytes, atypical cells, syncytia, and some mononuclear cells expressed viral antigens (Figure 3). Regarding the number of virus antigen-labelled cells, the CSc animals and the groups found dead (FD and FDc) frequently had a higher score compared to the NCSc group (Supplementary File 4, Supplementary Figure 3). There was a significant difference in IHC- Sars-CoV-2-positive cells in the NCSc and FDc groups (*p* < 0.05). In other organs of the culled minks (NCSc, CSc and FDc), virus antigen was expressed in macrophages of all the examined spleens (*n* = 14) and lymph nodes (*n* = 10/14) and less frequently in other organs, such as small and large intestine (*n* = 4), eye (*n* = 4), cerebrum (*n* = 3), kidney (*n* = 1), and heart (*n* = 1) (Table 3).

**Table 3:** Summary of the number and percentage of naturally infected minks with SARS-CoV-2 protein expression in different tissues.

Organ	Cells SARS-CoV-2+	Group 1 FD *N* = 15 (%)	Group 2 NCSc *N* = 10 (%)	Group 3 CSc *N* = 10 (%)	Group 4 FDc *N* = 9** (%)
Nose	Respiratory epithelial cells	9/15 (60)	3/10 (30)	9/10 (90)	9/9 (100)
Trachea	Respiratory epithelial cells	3/15 (20)	1/10 (10)	3/10 (30)	5/8* (62)
Lung	Respiratory epithelial cells; pneumocytes, atypical cells, syncytia, mononuclear cells	9/15 (60)	4/10 (40)	9/10 (90)	9/9 (100)
Duodenum	Macrophages	2/8* (25)	1/10 (10)	1/9* (10)	0/9 (0)
Colon	Macrophages	0/10* (0)	1/10 (10)	2/8* (25)	1/7* (14)
Lymph nodes	Macrophages	NA	4/4 (100)	4/5 (80)	2/5 (40)
Cerebrum	Macrophages	NA	1/6 (20)	0/5 (0)	2/6 (33)
Spleen	Macrophages	NA	4/4 (100)	5/5 (100)	5/5 (100)
Liver	–	NA	0/4 (0)	0/5 (0)	0/5 (0)
Heart	Macrophages	NA	0/4 (0)	0/5 (0)	1/5 (20)
Kidney	Macrophages	NA	0/4 (0)	0/5 (0)	1/5 (20)
Skin	–	NA	0/4 (0)	0/5 (0)	0/5 (0)
Tonsil	–	NA	0/4 (0)	0/5 (0)	0/5 (0)
Eye	Macrophages	NA	0/4 (0)	1/5 (20)	3/5 (60)

FD(C), found dead before and during culling; NCSc, no clinical signs culling; CSc, clinical signs culling.

*Tissue not present in all slides; **one autolytic case.

### Virology SARS-CoV-2 and ADV

**Figure 4:**
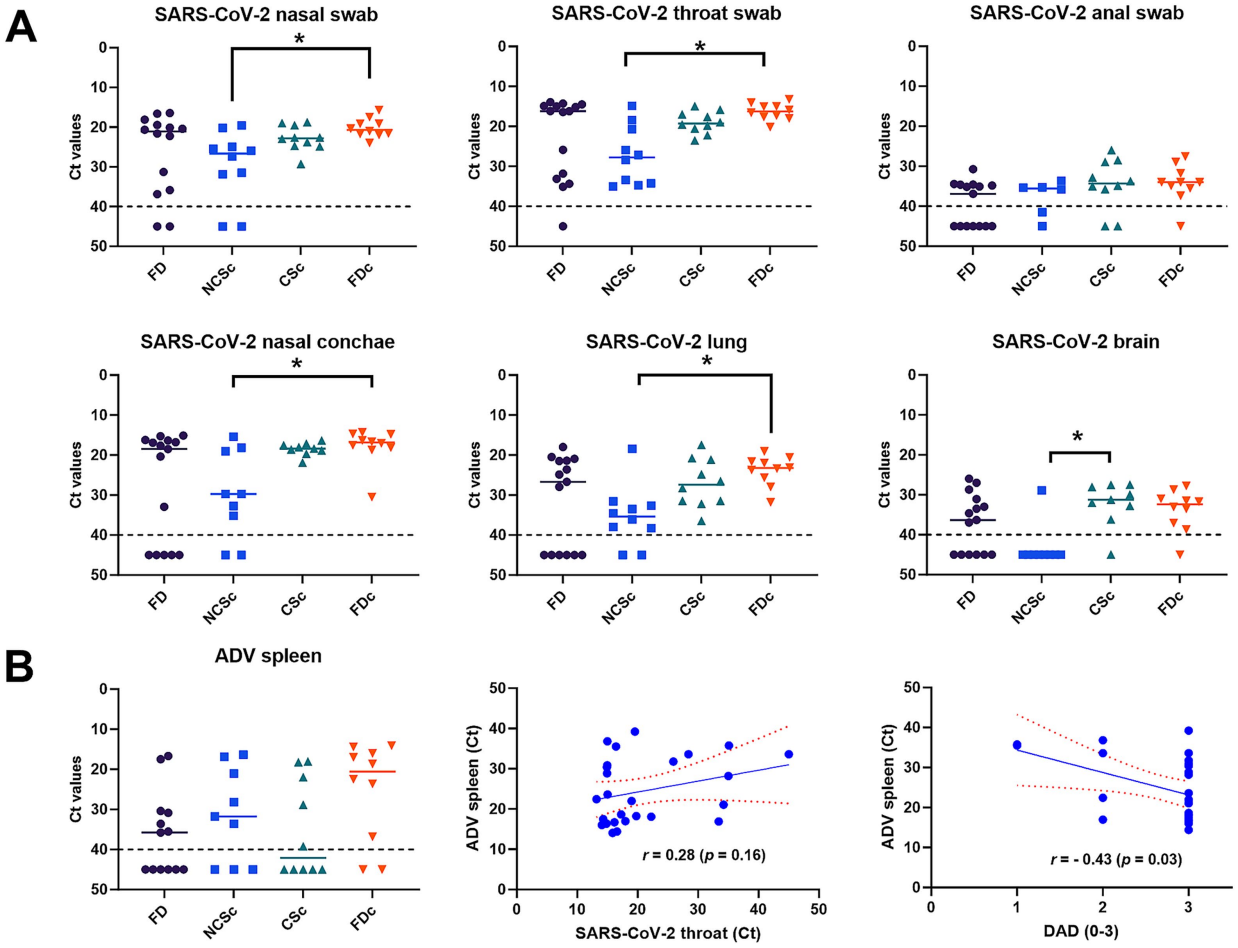
Viral RNA analysis in swabs and tissues **(A)** SARS-CoV-2 Ct values measured in swabs (nasal, throat and anal) and tissues (nasal conchae, lung and brain) in different groups of minks; **(B)** Aleutian disease (ADV), viral DNA in spleen and non-parametric Spearman correlation between ADV Ct value spleen and SARS-CoV-2 Ct value throat swab and severity score (0–3) diffuse alveolar damage (DAD) in lung; black dotted line is detection limit of qPCR, Ct values < 40 are considered positive; red dotted line is 95% confidence interval; FD(c), found dead before and during culling (c); NCSc, no clinical signs culling; CSc, Clinical signs culling; horizontal lines indicate the group average; significances are indicated by asterisk * *p* < 0.05.

From all animals across all groups, swabs were collected to detect SARS-CoV-2 RNA in the nose, throat, and rectum. Throat and nasal swabs of animals of group CSc contained significantly more viral RNA, as indicated by overall lower Ct values (Figure 4A), compared to group NCSc. There were no significant differences between the other groups, and no differences were observed between the groups for the anal swabs. Viral RNA was also measured in the lung, nasal conchae, and cerebrum and cerebellum in all animals in all groups. The lung tissue and nasal conchae of animals in group FDc contained significantly more viral RNA compared to group NCSc, and the brain of the CSc animals contained significantly more viral RNA compared to the NCSc animals. Additionally, viral RNA load was investigated in major organs and the intestine for a selection of animals sampled during the final culling on the farm (Figure 2). In the NCSc, viral RNA was detected only in respiratory organs and in one animal, as well as in the myocardium and spleen. The CSc animals also showed viral RNA in the brain, whereas in the FDc animals, viral RNA was detected in the intestine and various major organs. Phylogenetic analysis of SARS-CoV-2 sequences from throat swabs of randomly selected animals, both before and during culling, suggests 99.9% homology across groups (data not shown).

Because the mink farm has a history of ADV infection, which may have influenced the susceptibility to SARS-Cov-2 infection, the ADV status of the animals was measured by qPCR in the spleen of all animals (Figure 4B). There were no significant differences between the groups for the level of ADV DNA in the spleen, nor for the percentage of animals that tested positive for the presence of viral DNA (57.78% for all groups combined). Additionally, we showed no significant positive correlation between the SARS-CoV-2 throat swab Ct values and the ADV spleen Ct values; however, there was a significant negative association between DAD severity and ADV spleen Ct values (Figure 4B), indicating that, in our cohort of animals, higher viral ADV loads were associated with greater DAD severity.

We further compared associations between SARS-CoV-2 viral loads, IHC virus antigen scores and lung histopathology scores. A significant inverse association was observed between nasal SARS-CoV-2 Ct values and SARS-CoV-2 nasal antigen expression in IHC (Spearman *ρ* = −0.414, *p* < 0.05, FDR < 0.05), indicating increased antigen expression in samples with higher viral load. A similar association was observed in the lungs (ρ = −0.769, *p* < 0.0001, FDR < 0.0001), demonstrating that lower SARS-CoV-2 Ct values robustly tracked with increased viral antigen presence in the lung. However, no statistically significant associations were observed between SARS-CoV-2 lung viral load or antigen detection and histopathological severity scores after correction for multiple testing (Supplementary File 7a).

### Immunology: serology and proteomics

**Figure 5:**
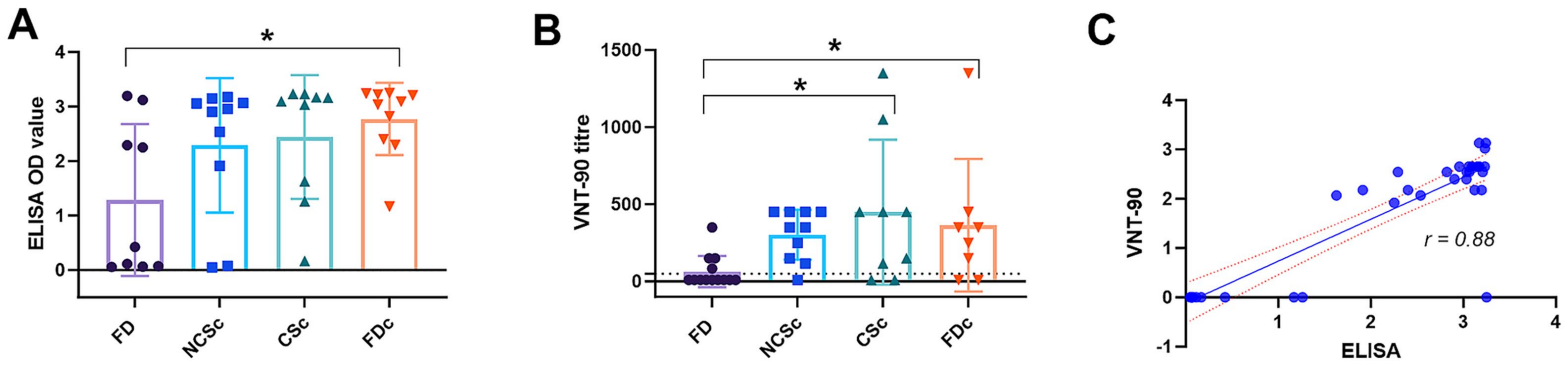
SARS-CoV-2 serology **(A)** SARS-CoV-2 RBD binding antibodies optical density (OD); **(B)** virus neutralizing antibodies VNT-90 titers, bar is group average and error bar shows SD; **(C)** Pearson correlation between ELISA (OD) and log10 VNT-90, dotted lines indicate 95% confidence interval; FD(c), Found dead before and during culling (c); NCSc, No clinical signs culling; CSc, Clinical signs culling; significances are indicated by asterisk * *p* < 0.05.

SARS-CoV-2 RBD binding antibodies were found in all groups (Figure 5A). The FD group had the most animals without specific antibodies. There was a significant difference in antibody levels between found dead animals before culling (FD) and during culling (FDc).

In addition to SARS-CoV-2-specific antibody titers, we assessed the capacity of serum antibodies to neutralize the SARS-CoV-2 virus. Overall, CSc and FDc animals showed higher VNT90 titres, with a significant difference between FD of both groups (Figure 5B). ELISA antibody OD values showed a significant correlation with the VNT titre (Figure 5C).

Blood samples from a selection of NCSc, CSc, and FDc animals were analyzed by Olink proteomics using an immunomodulation-oncology targeted biomarker panel. Principal component analysis (PCA) of normalized protein expression (NPX) values showed that FDc animals clustered closely together, suggesting lower within-group variability in protein expression compared to NCSc and CSc samples which exhibited a broader distribution. However, samples from the three groups did not segregate into separate clusters in the PCA and, similarly, unsupervised hierarchical clustering did not reveal any group-specific protein expression patterns (Supplementary Files 7b, 8).

## Discussion

In response to the detection of SARS-CoV-2 on mink farms in the Netherlands, parameters related to infection and disease were assessed. The study included minks found dead at the onset of the outbreak and animals sampled 4 days later during culling, including those found dead, as well as minks culled with clinical signs of SARS-CoV-2 infection and those that appeared clinically unaffected. This study demonstrated that, across the different stages of SARS-CoV-2 disease in farmed minks, (histo)pathological changes were consistent with previously reported findings in minks in Europe and the United States, as well as in humans (2, 6, 9, 19, 40). Regarding the stage of disease and lesions across the different clinical groups, the published literature provides some descriptive details of the staging framework for the exudative, proliferative, and fibrosing phases of DAD; however, it lacks clear stratification by clinical category. The different clinical groups investigated in this study: FD, NCSc, CSc, FDc reveal differences in the severity and frequency of lesions. FD likely represented the most severe progression of the disease, showing advanced acute pulmonary lesions and widespread virus antigen presence, consistent with end-stage disease. The CSc lies between FDc and NCSc, with lesions that reflect an active but not yet fatal stage of infection. Interestingly, as in humans, DAD was identified in animals without clinical signs, and DAD appears to develop early in the infection, even before the onset of clinical signs (20).

The macroscopic pattern of lesions recorded in this study revealed a pulmonary dark-red discoloration, histologically represented by areas with classic features of the acute exudative phases of DAD (e.g., pneumocyte loss and sloughing, hyaline membrane formation). Type II pneumocyte proliferation, atypical pneumocytes, and syncytia were the only elements of an initial subacute phase of DAD. On the other hand, features of fibrous and myofibroblastic proliferation, suggesting progression to a chronic phase, were not detected in the examined minks, whereas the literature only occasionally addresses this feature in mink (9). The absence of fibrotic changes in this study is clearly related to the stage of the disease. Unlike minks, human patients were commonly supported with drug therapy, oxygen therapy, and mechanical ventilation, which prolonged survival and prevented the disease from progressing to more severe stages.

Vasculitis and fibrin thrombi formation are frequently reported as a major feature of COVID-19 in humans (41), have been reported in experimentally infected minks (13, 42). These features have not been reported in naturally infected minks (2, 9) and were not detected in the current study, whereas in human patients, pulmonary and multiorgan thrombosis are major features of the disease and a factor associated with increased risk of death (41). Most likely, the molecular pathogenesis and related pathways after SARS-CoV-2 infection might differ in minks compared to humans.

Regarding viral antigen expression, similar to previous literature, IHC demonstrated positivity in bronchiolar epithelial cells, intra-alveolar macrophages, and pneumocytes. Virus antigen was also detected in macrophages in extra-pulmonary tissues, aligning with data from experimental infections, where the virus antigen co-localizes with activated macrophages throughout the animal’s body (42). Reported upper respiratory tract lesions vary between studies, with some describing mild chronic suppurative rhinitis in a few cases, while others describe this as the most common finding (2, 9). As in other studies, the major positive IHC was seen in sloughed-off and intact epithelial cells of the respiratory epithelium. No IHC positivity was seen in olfactory neurons, which has been rarely described before. No other reports mention positive IHC parenchymal cells in the brain of infected animals, which aligns with the results of the current study (8, 40, 43). The earlier literature predominantly reported SARS-COV-2 antigen localization in respiratory tissues (9), with less emphasis on extra-respiratory sites. The current study describes the rare presence of antigen in extrapulmonary sites, such as intestinal epithelial cells, and in scattered macrophages in multiple organs, including lymph nodes, spleen, heart, kidney, eye, and meninges.

As expected, the findings of the association and ordinal logistic regression models indicate a strong concordance between SARS-CoV-2 viral load and antigen IHC detection, particularly in lung tissue. However, histopathological severity was not directly associated with measures of SARS-CoV-2 viral burden, suggesting that lung damage can increase after viral clearance as observed in experimental hamster studies (44) and in human cases (45). As in the farm investigated in this study, infections with ADV have been regularly diagnosed on Dutch mink farms. An earlier study reported co-infection with ADV and SARS-CoV-2 in minks (9). In particular, in our study, foci of lymphoplasmacytic inflammation observed in various tissues, such as the kidney, liver, and heart, have been linked to ADV infection rather than SARS-CoV-2 infection. In these cases, ADV Ct-values were around or below 20 (ranging from 14.39 to 23.59). In total, 26 out of 45 animals in this study tested positive for ADV and we showed that minks with a higher ADV viral load in the spleen were moderately associated with greater DAD severity. The mechanism of this is unclear and we speculate that an impaired or more tolerogenic innate immune response due to the chronic ADV infection could have made the mink more susceptible to the SARS-CoV-2 infection itself. However, the number of observations is limited, and the relationship appears to be influenced, due to the number of animals, by a small number of cases, including clustered values. The observed correlation may therefore overestimate the true effect size of ADV influence on SARS-CoV-2-induced DAD; replication in a larger, more evenly distributed sample would be therefore advisable.

SARS-CoV-2 shedding and transmission occurred most likely by direct nose/mouth contact, whereas fecal shedding was most likely less important compared to direct contact (2). Animals without clinical signs (NCSc) likely already contributed to virus transmission; however, FD animals had more viral RNA in the nose and throat swabs, as well as in the respiratory organs (lungs, nasal conchae) and brain, compared to animals without clinical signs. In the FD animals, the virus was more disseminated throughout the whole body (spleen, liver, kidney, jejunum, and colon) compared to the clinically ill animals. This supports the finding that fecal shedding is mainly important at the final stage of the disease. Unfortunately, we were unable to trace the source and date of the initial infection on this farm; however, it is most likely related to a human introduction, as previously described (15).

All minks developed a specific antibody response during culling, regardless of disease stage. In contrast, only 50% of the animals collected on 1st November were positive in the VNT, which is most likely related to an earlier stage of infection or the fact that the animals were not infected with SARS-CoV-2. Although all minks developed neutralizing antibodies, these were insufficient to prevent disease or mortality. The proteomics results indicated no difference between the clinical groups for immune-related proteins, consistent with observations of interstitial pneumonia, which was observed in all animals. However, the FDc animals clustered more tightly together, suggesting less variation in protein expression in this group. The applied proteomics technique was not validated for mink, and whole frozen blood was used for analysis; this could also lead to a lack of differences. However, previous research has shown cross-reactivity between human and mink protein samples (46).

This study, which investigated disease stages during culling at a mink farm, adds depth to understanding of SARS-CoV-2 pathology in minks and highlights specific patterns that mirror those of severe human COVID-19. In addition to the valuable data, our research has several limitations. The lack of data on disease progression in culled groups may limit the ability to generate hypotheses about disease progression in naturally infected minks. However, the fact that even in found dead animals, the lesions were in the acute stage of DAD suggests a possible rapid progression of the disease in this species, which could be related to the more prominent ACE2 distribution in the lower respiratory tract in mink, compared to ferrets and humans (12). Next to the species difference (47), the lack of features in the fibrotic stage of the disease could also be explained by the limited time available after initial infection, due to culling. Or it cannot be excluded that mink do not develop this final fibrotic stage. In experimentally infected ferrets, there was no documentation of this fibrotic stage at 21 days after infection (48); however, in the ferrets examined in literature, necrosis of alveolar septa was not detected, and lesions were generally reported to be of mild to moderate severity, possibly justifying the absence of a progression to a fibrotic stage of the disease.

Additionally, a significant finding from this study is the presence of high viral loads in throat and nasal swabs from animals that do not exhibit any signs of disease; therefore, they are considered clinically healthy. These findings suggest the potential of zoonotic virus transmission even in minks with subclinical disease and underscore the importance of relying not only on clinical observation but also on frequent sampling and molecular testing to detect outbreaks at an early stage. In addition to sampling, operators on mink farms should be aware of the zoonotic potential of SARS-CoV-2 and avian influenza (49). Operators’ age and potential underlying health conditions may indicate that they should wear appropriate face masks (50).

In conclusion, farmed minks naturally infected with SARS-CoV-2 demonstrated lung pathology similar to that described in humans during the acute phase of the disease, with lung changes extending farther and being more severe in dead and clinically ill minks than in those without clinical signs. Histopathological characteristics commonly observed in human diseases, such as vasculitis, thrombosis, and fibrosis, were absent in the lungs of the minks. The pathological changes in respiratory and extra-respiratory tissues were associated with the presence of virus antigen, as detected by IHC, and viral RNA in tissues. The presence of antibodies in serology indicated that the animals were infected at least 1 or 2 weeks before culling. The exact mechanism and role of co-infection with SARS-CoV-2 in chronically infected ADV minks remain unclear; however in this study, the presence of ADV viral DNA seemed to increase the severity of DAD in SARS-CoV-2 infected lungs. SARS-CoV-2 infected but clinically healthy farmed minks showed severe lung lesions with high viral loads in different organs, suggesting the potential of severe disease and virus transmission even in animals not showing any signs of disease.

## Data Availability

The datasets presented in this study can be found in online repositories. The names of the repository/repositories and accession number(s) can be found in the article/Supplementary material.
