# Supplementary material for: Natural SARS-CoV-2 infection in farmed minks (Neovison vison) causes lung pathology, systemic viral spread, and transmission risk, even in asymptomatic animals
Source: Front Vet Sci. 2026 Mar 24;13:1752459. doi: 10.3389/fvets.2026.1752459 (PMC13054983; doi:10.3389/fvets.2026.1752459)
Supplement: Supplementary file 2 [file Supplementary_file_2.docx]

**Supplementary File 2 Macroscopic findings**

**Table**: Summary of the main macroscopic findings in SARS-CoV-2 naturally infected minks.

| **Organ** | **Lesions** | **Group 1**  **FD**  **N=15 (%)** | **Group 2**  **NCSc**  **N=10 (%)** | **Group 3**  **CSc**  **N=10 (%)** | **Group 4**  **FDc**  **N = 10 (%)** |
| --- | --- | --- | --- | --- | --- |
| **Nasal cavity** | Mucopurulent rhinitis | 0/15 (0) | 2/10 (20) | 5/10 (50) | 6/10 (60) |
| **Trachea** | Foam | 0/15 (0) | 1/10 (10) | 2/10 (20) | 0/10 (0) |
| **Tracheo-bronchial lymph node** | Lymphadenomegaly | 5/15 (30) | 9/10 (90) | 6/10 (60) | 6/10 (60) |
| **Lungs** | Diffuse dark-red discoloration, oedema, consolidation | 11/15 (73) | 6/10 (60) | 7/10 (70) | 9/10 (90) |
| **Heart** | Pericardial effusion    Ventricular dilation | 0/15 (0)    0/15 (0) | 0/10 (0)    1/10 (10) | 0 / 10 (0)    1 / 10 (10) | 1/10 (10)    0/10 (0) |
| **Liver** | Pallor    Hepatomegaly | 2/15 (13)    3/15 (20) | 0/10 (0)    1/10 (10) | 1 / 10 (10)    3 / 10 (30) | 0/10 (0)    0/10 (0) |
| **Spleen** | Splenomegaly | 2/15 (13) | 3/10 (30) | 4/10 (40) | 2/10 (20) |

FD(c) = Found dead before and during culling, NCSc = No clinical signs, CSc = clinical signs;


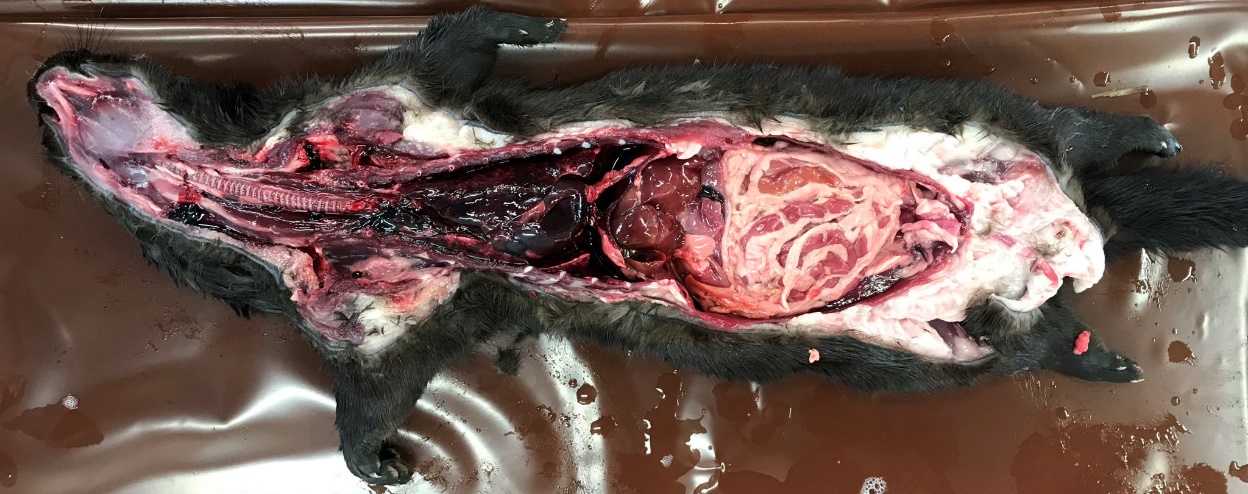


**Figure 1 Macroscopy**: In situ overview of mink #8 (group 1 found dead (FD))


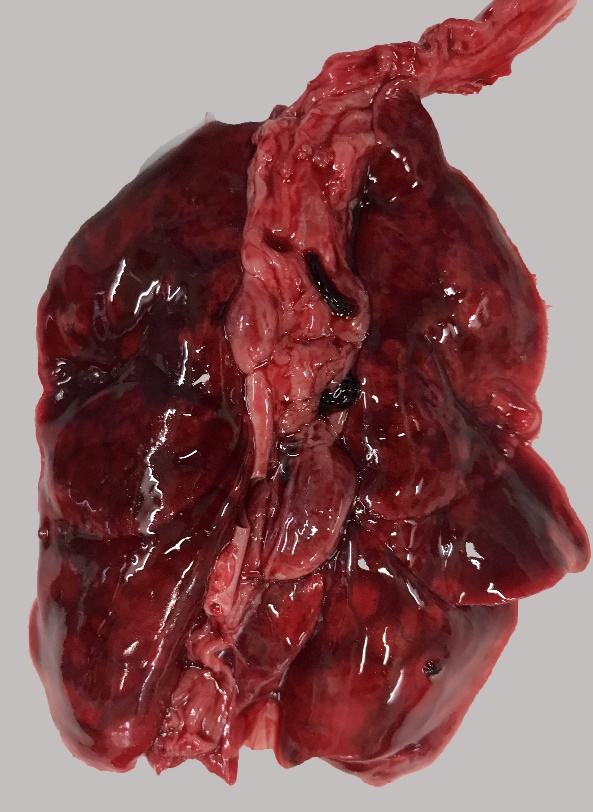

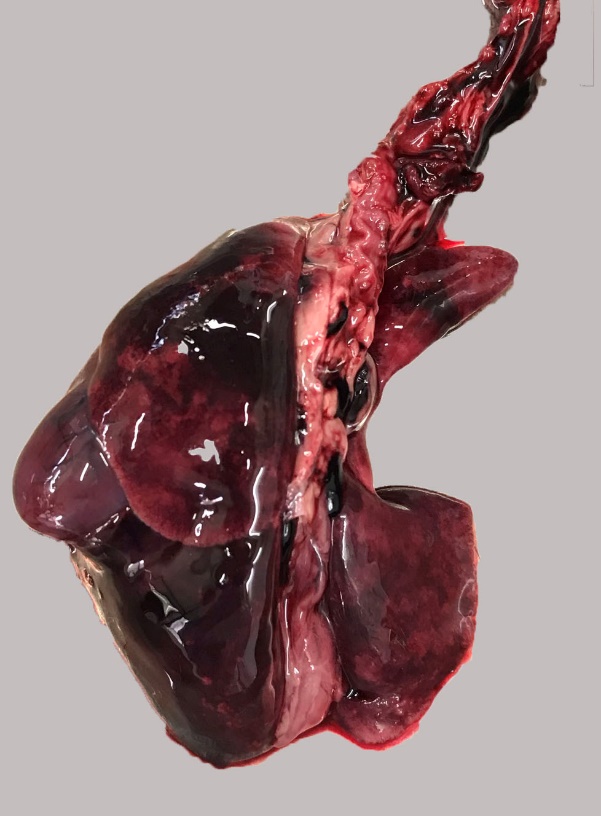


**B**

**A**

**Figure 2 Macroscopy**: Dorsal view of lungs **(A)** group 1 found dead (FD) mink #8 ;**(B)** group 3 clinically ill mink (CSc) #29. Diffuse patchy dark-red discoloration with edema.
