# Supplementary material for: Natural SARS-CoV-2 infection in farmed minks (Neovison vison) causes lung pathology, systemic viral spread, and transmission risk, even in asymptomatic animals
Source: Front Vet Sci. 2026 Mar 24;13:1752459. doi: 10.3389/fvets.2026.1752459 (PMC13054983; doi:10.3389/fvets.2026.1752459)
Supplement: Supplementary file 3 [file Supplementary_file_3.docx]

**Supplementary File 3** Data on the pathology scoring of major pulmonary findings in minks.

| **ID** | **Cat.** | **DAD** | **PN** | **TR** | **RN** | **ALT** | **AO** | **HM** | **AH** | **TpII H** | **AtP** | **FOR** | **SYN** | **mT** |
| --- | --- | --- | --- | --- | --- | --- | --- | --- | --- | --- | --- | --- | --- | --- |
| 1 | FD | mild | mild | n | mild | mild | mild | - | - | - | - | - | - | - |
| 2 | FD | moderate | mild | moderate | mild | moderate | mild | - | - | - | - | - | - | - |
| 3 | FD | mild | mild | mild | - | mild | mild | - | - | - | - | - | - | - |
| 4 | FD | mild | mild | mild | - | mild | mild | - | - | - | - | - | - | - |
| 5 | FD | moderate | mild | - | - | moderate | mild | - | - | - | - | - | - | - |
| 6 | FD | severe | moderate | - | severe | severe | mild | mild | moderate | mild | moderate | - | mild | - |
| 7 | FD | severe | moderate | mild | severe | severe | moderate | - | moderate | - | - | - | - | - |
| 8 | FD | severe | severe | mild | severe | severe | mild | mild | moderate | - | mild | - | - | - |
| 9 | FD | severe | mild | mild | severe | severe | moderate | - | moderate | - | - | - | mild | - |
| 10 | FD | severe | severe | mild | moderate | severe | moderate | - | moderate | - | mild | - | mild | - |
| 11 | FD | severe | mild | mild | severe | severe | moderate | mild | moderate | - | - | - | mild | - |
| 12 | FD | severe | mild | mild | moderate | severe | moderate | - | moderate | - | mild | - | mild | - |
| 13 | FD | severe | severe | mild | severe | severe | moderate | moderate | moderate | - | severe | - | mild | - |
| 14 | FD | Mild | mild | mild | severe | mild |  | - | - | - | - | - | - | - |
| 15 | FD | severe | moderate | n | moderate | severe | moderate | - | moderate | - | - | - | mild | - |
| 16 | NCSc | mild | n | n | n | mild | mild | - | moderate | - | - | - | - | - |
| 17 | NCSc | severe | moderate | mild | mild | severe | mild | - | mild | - | mild | - | mild | - |
| 18 | NCSc | severe | moderate | mild | severe | severe | mild | - | moderate | - | - | - | mild | - |
| 19 | NCSc | severe | severe | n | mild | severe | moderate | - | severe | - | mild | - | mild | - |
| 20 | NCSc | severe | moderate | n | moderate | severe | moderate | - | moderate | - | mild | - | mild | - |
| 21 | NCSc | severe | mild | n | mild | severe | moderate | - | moderate | - | severe | - | moderate | - |
| 22 | NCSc | severe | severe | mild | mild | severe | mild | - | moderate | - | moderate | - | moderate | - |
| 23 | NCSc | severe | moderate | n | mild | severe | moderate | - | severe | mild | severe | - | moderate | - |
| 24 | NCSc | severe | mild | n | mild | severe | mild | - | moderate | - | - | - | mild | - |
| 25 | NCSc | severe | mild | n | n | severe | mild | - | moderate | - | mild | - | mild | - |
| 26 | CSc | severe | mild | mild | moderate | severe | mild | - | severe | - | - | - | moderate | - |
| 27 | CSc | severe | moderate | mild | moderate | severe | moderate | - | mild | - | severe | - | severe | - |
| 28 | CSc | severe | mild | severe | severe | severe | moderate | - | mild | - | moderate | - | mild | - |
| 29 | CSc | severe | moderate | mild | severe | severe | moderate | - | mild | - | moderate | - | mild | - |
| 30 | CSc | severe | mild | mild | moderate | severe | moderate | - | mild | - | moderate | - | mild | - |
| 31 | CSc | severe | mild | n | moderate | severe | moderate | - | mild | - | severe | - | mild | - |
| 32 | CSc | moderate | mild | mild | moderate | moderate | mild | - | mild | - | severe | - | mild | - |
| 33 | CSc | severe | moderate | mild | moderate | severe | moderate | - | mild | - | moderate | - | mild | - |
| 34 | CSc | severe | moderate | mild | severe | severe | moderate | - | mild | - | moderate | - | moderate | - |
| 35 | CSc | severe | n | mild | mild | severe | mild | - | mild | - | - | - | - | - |
| 36 | FDc | severe | mild | moderate | severe | severe | moderate | moderate | - | - | mild | - | mild | - |
| 37 | FDc | severe | mild | mild | severe | severe | moderate | moderate | - | - | mild | - | mild | - |
| 38 | FDc | severe | severe | n | moderate | severe | severe | severe | mild | - | severe | - | severe | - |
| 39 | FDc | severe | mild | moderate | severe | severe | moderate | severe | mild | - | moderate | - | mild | - |
| 40 | FDc | severe | mild | mild | moderate | severe | moderate | moderate | severe | - | mild | - | mild | - |
| 41 | FDc | severe | severe | moderate | moderate | severe | moderate | severe | moderate | - | mild | - | severe | - |
| 42 | FDc | moderate | mild | mild | moderate | moderate | mild | mild | mild | - | mild | - | mild | - |
| 43 | FDc | moderate | mild | mild | severe | moderate | moderate | mild | mild | - | mild | - | mild | - |
| 44 | FDc | autolysis | - | - | - | autolysis | - | - | - | - | - | - | - | - |
| 45 | FDc | moderate | mild | mild | mild | moderate | - | mild | mild | - | mild | - | - | - |

Cat. = Category; FD = Found dead; NCSc = no clinical signs culled; CSc = Clinical sign culled; FDc = Found dead culled; CC = cell count; G = grade; DAD = diffuse alveolar damage; PN = pneumonia; TR = tracheitis; RN = rhinitis; ALT = alveolitis; AO = alveolar oedema; HM = hyaline membranes; AH = alveolar hemorrhage; TpII H = type II pneumocytes hyperplasia; AtP = atypical pneumocytes; FOR = fibrosis-organising stage; SYN = syncytia; mT = microthrombi.
