# Supplementary material for: Natural SARS-CoV-2 infection in farmed minks (Neovison vison) causes lung pathology, systemic viral spread, and transmission risk, even in asymptomatic animals
Source: Front Vet Sci. 2026 Mar 24;13:1752459. doi: 10.3389/fvets.2026.1752459 (PMC13054983; doi:10.3389/fvets.2026.1752459)
Supplement: Supplementary file 4 [file Supplementary_file_4.docx]

**Supplementary File 4**

**Figure 1, 2 and 3**


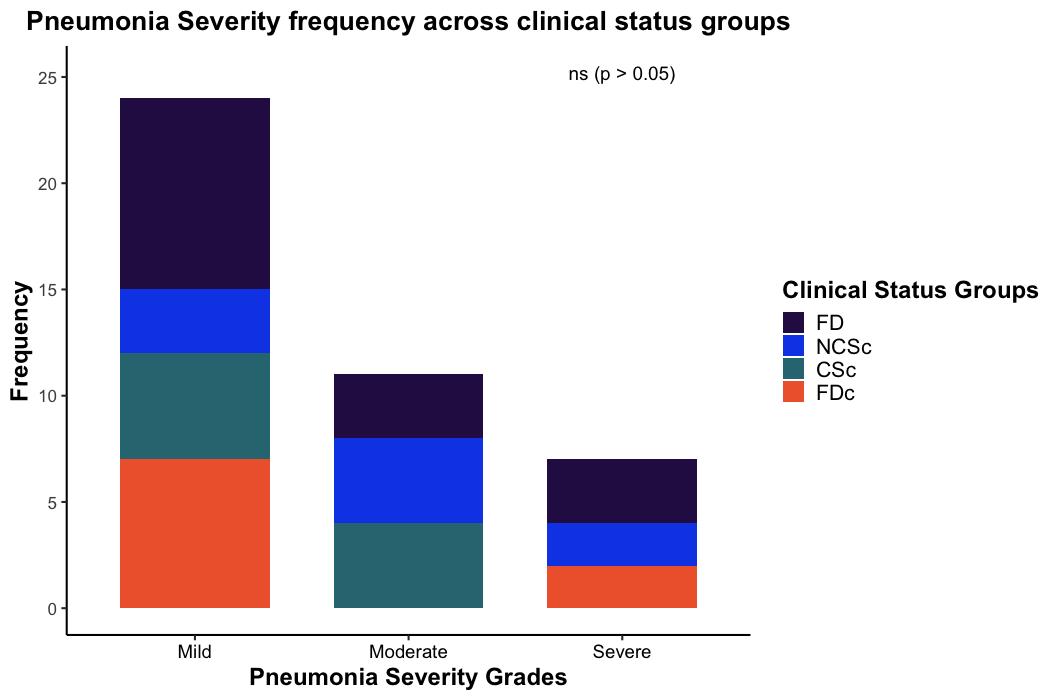


**Supplementary Figure 1.** Relative frequencies of the pneumonia severity grades (Grade 1 - Mild, Grade 2 - Moderate, Grade 3 - Severe) assessed on histology across the different clinical groups (FD n =15, NCSc n = 10, CS n = 10, FDc n = 9**). No significant (ns) association of the pneumonia severity grades with the clinical groups was detected at the Chi-square test (p > 0.05); ** one autolytic case; FD = Found Dead, NCSc = No Clinical Signs culling, CSc = Clinically Signs culling, FDc = Found Dead at Culling.


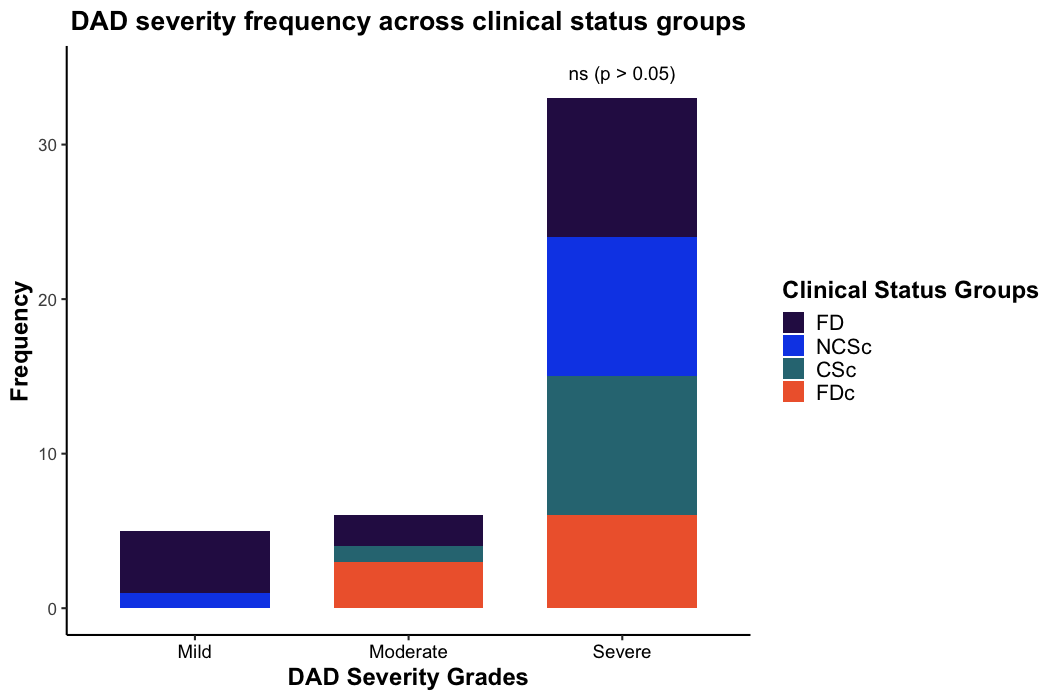


**Supplementary Figure 2.** Relative frequencies of the diffuse alveolar damage (DAD) severity grades (Grade 1 - Mild, Grade 2 - Moderate, Grade 3 - Severe) assessed on histology across the different clinical groups (FD n =15, NCSc n = 10, CS n = 10, FDc n = 9**). No significant (ns) association of the diffuse alveolar damage severity grades with the clinical groups was detected at the Chi-square test (p > 0.05); ** one autolytic case; FD = Found Dead, NCSc = No Clinical Signs culling, CSc = Clinically Signs culling, FDc = Found Dead at Culling.


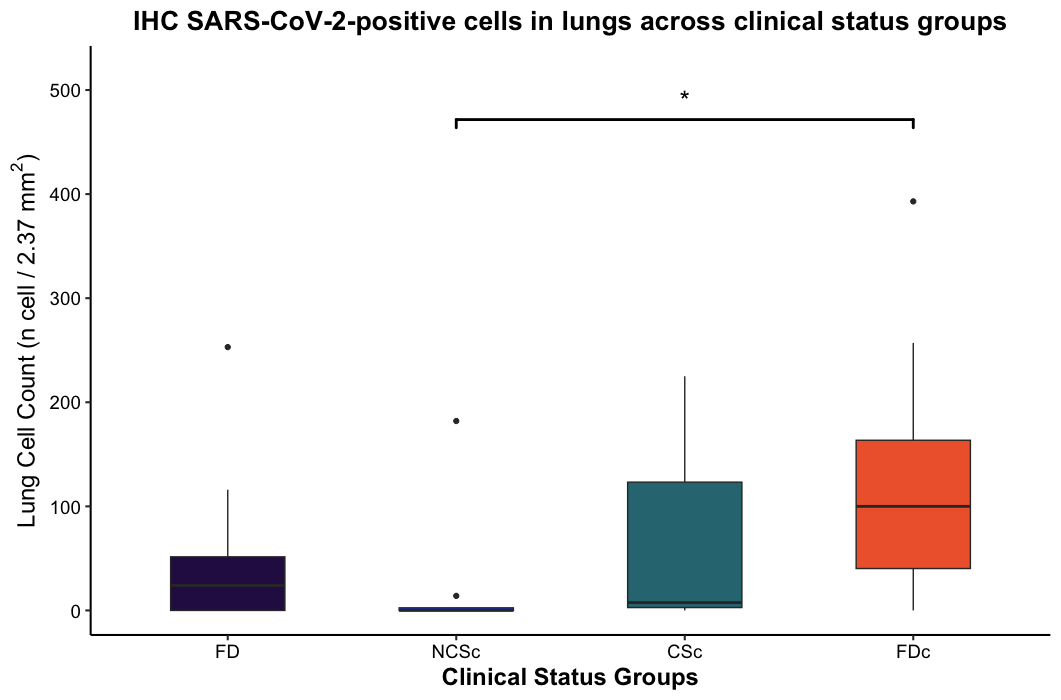


**Supplementary Figure 3.** SARS-CoV-2-positive cell count in the lung assessed on immunohistochemistry across clinical groups (n cell / 2.37 mm^2^ tissue area) with a different clinical status (FD n =15, NCSc n = 10, CS n = 10, FDc n = 9**) of SARS-CoV-2 naturally infected minks. A significant difference in IHC- SARS-CoV-2-positive cells was detected in the NCSc and FDc groups at Kruskal-Wallis test and Dunn's test (p < 0.05; *);

** one autolytic case; FD = Found Dead, NCSc = No Clinical Signs culling, CSc = Clinically Signs culling, FDc = Found Dead at Culling.
