# Supplementary material for: Natural SARS-CoV-2 infection in farmed minks (Neovison vison) causes lung pathology, systemic viral spread, and transmission risk, even in asymptomatic animals
Source: Front Vet Sci. 2026 Mar 24;13:1752459. doi: 10.3389/fvets.2026.1752459 (PMC13054983; doi:10.3389/fvets.2026.1752459)
Supplement: Supplementary file 5 [file Supplementary_file_5.docx]

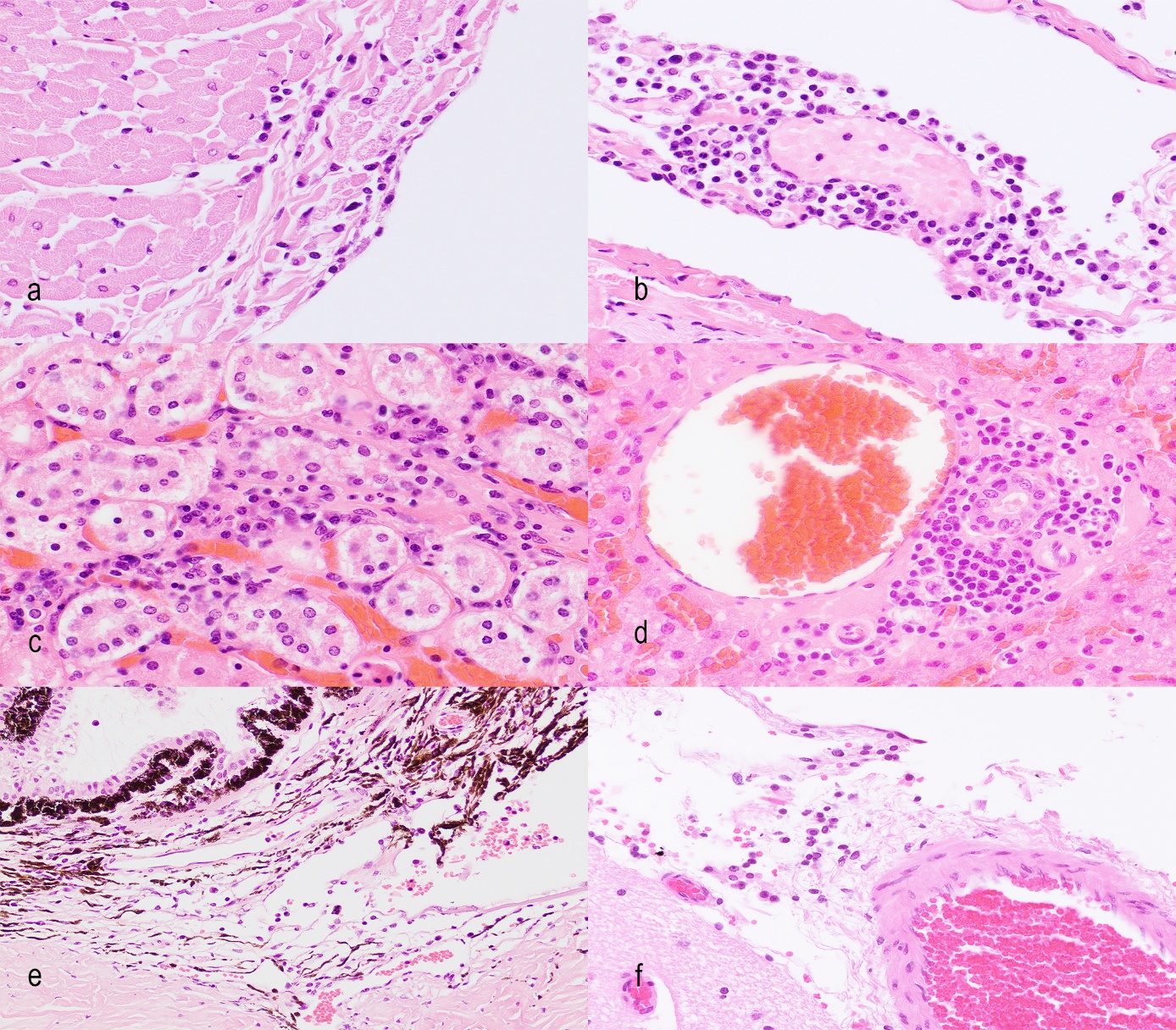
**Supplementary File 5**

**Histopathology of extra-respiratory organs and tissues of SARS-CoV-2 naturally infected minks and ADV-related changes**.

a) Epicardium and myocardium showing a mild lymphoplasmacytic infiltrate. Hematoxylin and eosin (HE; x400). b) Epicardium showing a moderate perivascular lymphoplasmacytic infiltrate (HE; x400). c) Renal interstitium showing a mild lymphoplasmacytic infiltrate (HE; x400). d) Hepatic portal area showing a moderate lymphoplasmacytic infiltrate surrounding biliary ducts (#HE; x400). e) Ocular drainage angle showing a mild lymphoplasmacytic infiltrate and a low number of extravasated erythrocytes (HE; x400). f) Meninges and neuroparenchyma showing a mild lymphoplasmacytic infiltrate, a low number of extravasated erythrocytes (HE; x400).
