# Supplementary material for: Natural SARS-CoV-2 infection in farmed minks (Neovison vison) causes lung pathology, systemic viral spread, and transmission risk, even in asymptomatic animals
Source: Front Vet Sci. 2026 Mar 24;13:1752459. doi: 10.3389/fvets.2026.1752459 (PMC13054983; doi:10.3389/fvets.2026.1752459)
Supplement: Supplementary file 6 [file Supplementary_file_6.docx]

**Supplementary File 6 : Table** Data on the immunohistochemical Sars-CoV-2 positive cell count and grade in different respiratory sites and cells.

| **ID** | **Cat.** | **Nose CC** | **Nose G** | **OEp** | **Trachea CC** | **Trachea G** | **Lung CC** | **Lung G** | **Lung BEp CC** | **Lung tpI CC** | **Lung alv.mac. CC** | **Lung other cells CC** |
| --- | --- | --- | --- | --- | --- | --- | --- | --- | --- | --- | --- | --- |
| 1 | FD | 0 | 0 | N | 0 | 0 | 0 | 0 | 0 | 0 | 0 | 0 |
| 2 | FD | 0 | 0 | N | 0 | 0 | 0 | 0 | 0 | 0 | 0 | 0 |
| 3 | FD | 0 | 0 | N | 0 | 0 | 5 | 1 | 0 | 1 | 1 | 0 |
| 4 | FD | 0 | 0 | N | 0 | 0 | 0 | 0 | 0 | 0 | 0 | 0 |
| 5 | FD | 0 | 0 | N | 0 | 0 | 0 | 0 | 0 | 0 | 0 | 0 |
| 6 | FD | 251 | 3 | N | 0 | 0 | 24 | 1 | 2 | 1 | 1 | 0 |
| 7 | FD | 119 | 2 | N | 0 | 0 | 253 | 3 | 1 | 3 | 2 | 0 |
| 8 | FD | 198 | 2 | N | 5 | 1 | 56 | 1 | 2 | 1 | 1 | 0 |
| 9 | FD | 30 | 1 | N | 0 | 0 | 0 | 0 | 0 | 0 | 0 | 0 |
| 10 | FD | 152 | 2 | N | 0 | 0 | 45 | 1 | 1 | 2 | 2 | 1 |
| 11 | FD | 327 | 3 | N | 3 | 1 | 45 | 1 | 2 | 1 | 1 | 0 |
| 12 | FD | 243 | 3 | N | 0 | 0 | 47 | 1 | 1 | 2 | 2 | 0 |
| 13 | FD | 130 | 2 | N | 113 | 2 | 116 | 2 | 2 | 2 | 1 | 0 |
| 14 | FD | 0 | 0 | N | 0 | 0 | 0 | 0 | 0 | 0 | 0 | 0 |
| 15 | FD | 231 | 3 | N | 0 | 0 | 80 | 1 | 0 | 3 | 1 | 0 |
| 16 | NCSc | 0 | 0 | N | 0 | 0 | 0 | 0 | 0 | 0 | 0 | 0 |
| 17 | NCSc | 0 | 0 | N | 0 | 0 | 0 | 0 | 0 | 0 | 0 | 0 |
| 18 | NCSc | 405 | 3 | N | 25 | 1 | 182 | 2 | 1 | 3 | 2 | 1 |
| 19 | NCSc | 0 | 0 | N | 0 | 0 | 0 | 0 | 0 | 0 | 0 | 0 |
| 20 | NCSc | 100 | 1 | N | 0 | 0 | 14 | 1 | 1 | 1 | 1 | 0 |
| 21 | NCSc | 62 | 1 | Y | 0 | 0 | 1 | 1 | 0 | 1 | 0 | 0 |
| 22 | NCSc | 0 | 0 | N | 0 | 0 | 0 | 0 | 0 | 0 | 0 | 0 |
| 23 | NCSc | 0 | 0 | N | 0 | 0 | 3 | 1 | 0 | 1 | 0 | 0 |
| 24 | NCSc | 0 | 0 | N | 0 | 0 | 0 | 0 | 0 | 0 | 0 | 0 |
| 25 | NCSc | 0 | 0 | N | 0 | 0 | 0 | 0 | 0 | 0 | 0 | 0 |
| 26 | CSc | 33 | 1 | N | 0 | 0 | 225 | 3 | 1 | 3 | 2 | 1 |
| 27 | CSc | 107 | 2 | N | 0 | 0 | 94 | 1 | 1 | 3 | 2 | 1 |
| 28 | CSc | 28 | 1 | N | 0 | 0 | 5 | 1 | 0 | 1 | 1 | 0 |
| 29 | CSc | 45 | 1 | N | 23 | 1 | 133 | 2 | 1 | 3 | 2 | 1 |
| 30 | CSc | 123 | 2 | N | 3 | 1 | 1 | 1 | 0 | 1 | 0 | 0 |
| 31 | CSc | 35 | 1 | N | 0 | 0 | 10 | 1 | 0 | 1 | 1 | 0 |
| 32 | CSc | 66 | 1 | Y | 0 | 0 | 2 | 1 | 0 | 1 | 1 | 0 |
| 33 | CSc | 448 | 3 | N | 0 | 0 | 163 | 2 | 1 | 3 | 2 | 1 |
| 34 | CSc | 89 | 1 | Y | 4 | 1 | 5 | 1 | 0 | 1 | 1 | 0 |
| 35 | CSc | 0 | 0 | N | 0 | 0 | 0 | 0 | 0 | 0 | 0 | 0 |
| 36 | FDc | 133 | 2 | N | 64 | 1 | 50 | 1 | 2 | 1 | 2 | 0 |
| 37 | FDc | 44 | 1 | Y | 1 | 1 | 37 | 1 | 0 | 2 | 2 | 0 |
| 38 | FDc | 196 | 2 | Y | 0 | 0 | 117 | 2 | 3 | 2 | 2 | 1 |
| 39 | FDc | 94 | 1 | N | 5 | 1 | 83 | 1 | 2 | 2 | 2 | 0 |
| 40 | FDc | 157 | 2 | N | 0 | 0 | 179 | 2 | 2 | 3 | 2 | 0 |
| 41 | FDc | 100 | 1 | N | 229 | 3 | 257 | 3 | 2 | 3 | 2 | 2 |
| 42 | FDc | 258 | 3 | N | 145 | 2 | 117 | 2 | 3 | 2 | 2 | 0 |
| 43 | FDc | 36 | 1 | N | 0 | 0 | 33 | 1 | 0 | 1 | 1 | 0 |
| 44 | FDc | 17 | 1 | N | 0 | 0 | 0 | 0 | 0 | 0 | 0 | 0 |
| 45 | FDc | 251 | 3 | N | 0 | 0 | 393 | 3 | 2 | 3 | 2 | 0 |

Cat. = Category; FD = Found dead; NCSc = no clinical signs culled; CSc = Clinical sign culled; FDc = Found dead culled; CC = cell count; G = grade; OEp = olfacrory epithelium; BEp = bronchial epithelium; tpI = type I pneumocytes; alv.mac. = alveolar macrophages
