# Supplementary material for: Natural SARS-CoV-2 infection in farmed minks (Neovison vison) causes lung pathology, systemic viral spread, and transmission risk, even in asymptomatic animals
Source: Front Vet Sci. 2026 Mar 24;13:1752459. doi: 10.3389/fvets.2026.1752459 (PMC13054983; doi:10.3389/fvets.2026.1752459)
Supplement: Supplementary file 7 [file Supplementary_file_7.docx]

**Supplementary File 7**

**Part 7a**

**Additional information results section “ Virology SARS-CoV-2 and ADV”: Association of virology and histology**

No statistically significant associations were observed between lung viral load or antigen detection and histopathological severity scores after correction for multiple testing (Supplementary file 2a). Specifically, neither the histological pneumonia severity score nor the diffuse alveolar damage score was significantly correlated with SARS-CoV-2 lung Ct values or lung IHC scores (all FDR-adjusted p-values > 0.05). In an ordinal logistic regression model including lung SARS-CoV-2 viral antigen amount in IHC and lung Ct value, each one-unit increase in lung Ct was associated with a 36% reduction in the odds of higher lung IHC scores (OR = 0.64, 95% CI 0.51–0.80, p < 0.0001). In contrast, neither lung Ct nor lung IHC was independently associated with pneumonia severity or diffuse alveolar damage scores after adjustment in multivariable ordinal models (p > 0.05). Proportional odds assumptions were met.

**
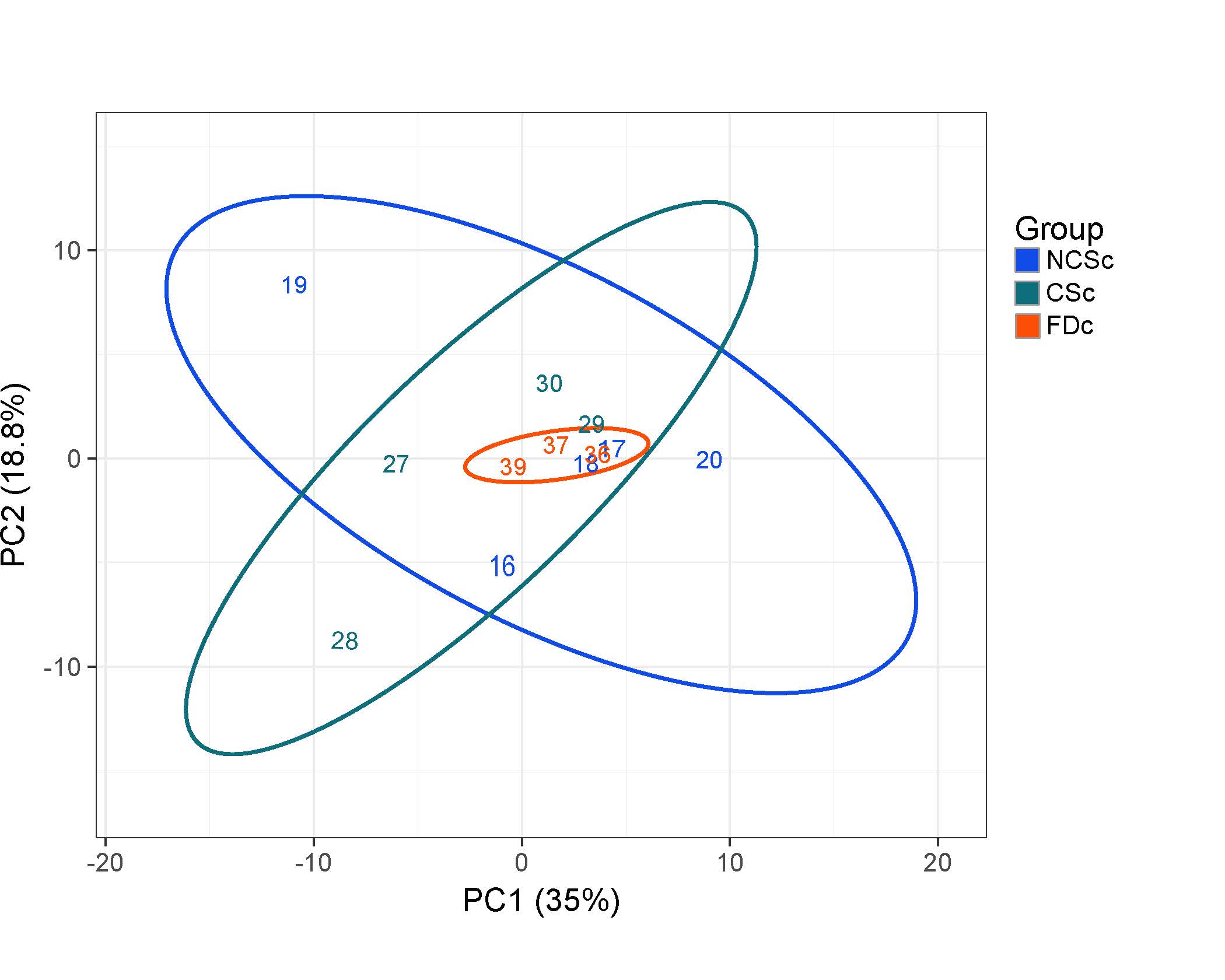
Supplementary File 7**

**Part 7b: Proteomics results**

**Supplementary Figure 1: Principal component analysis based on protein NPX values (transformed to linear scale). Olink proteomics was performed** using whole blood samples of 12 culled minks: animals without clinical signs (NCSc, n=5, #16, 17, 18, 19 and 20), with clinical signs (CSc, n=4, #27, 28, 29 and 30), and found dead (FDc, n=3, #36, 37 and 39 ). SVD with imputation was used to calculate principal components. The X and Y axes represent principal components 1 and 2, which account for 35% and 18.8% of the total variance in the dataset, respectively. Prediction ellipses indicate that with a probability of 0.95, a new observation from the same group will fall inside the ellipse.


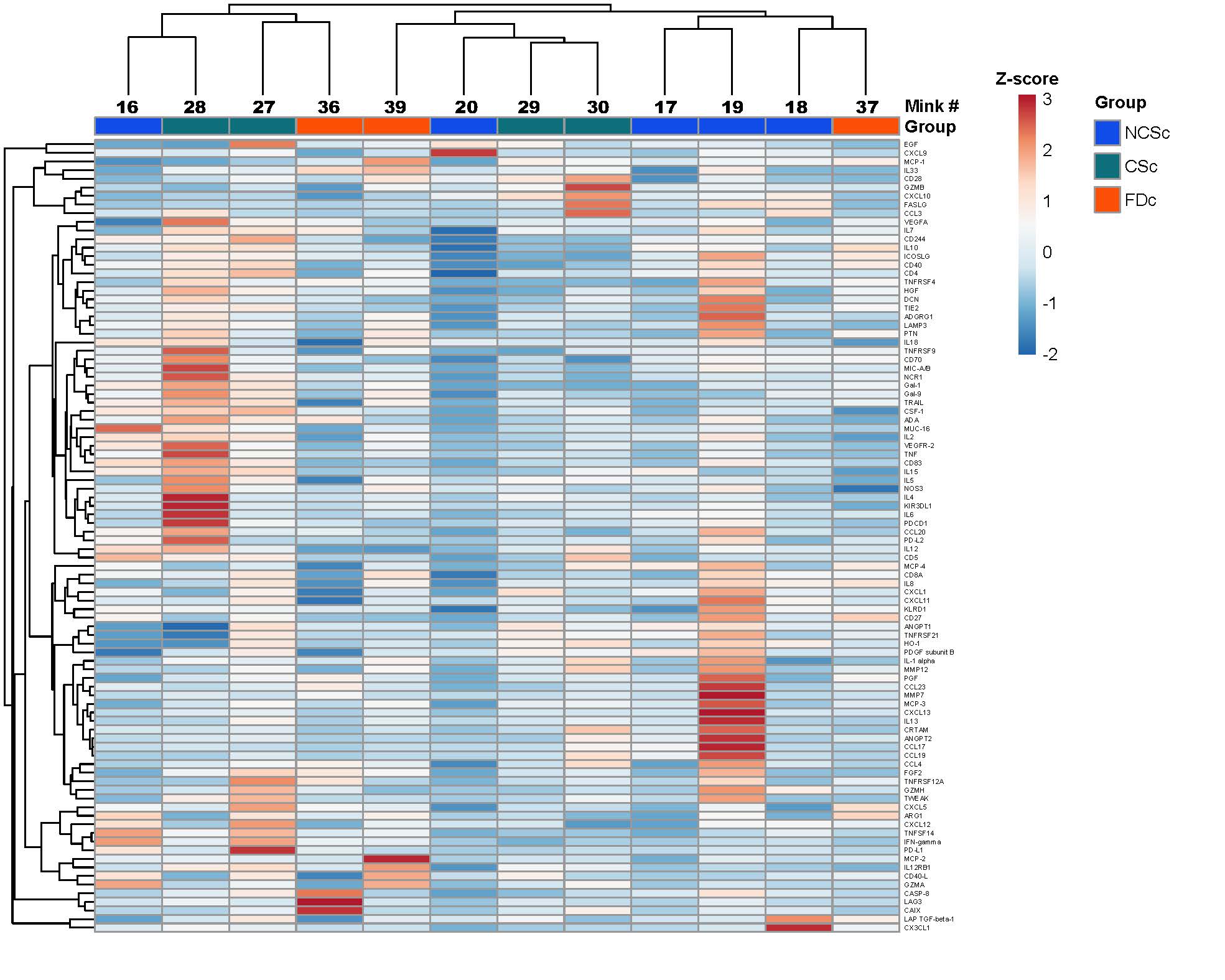


**Supplementary Figure 2 Heatmap of immuno-oncology related biomarkers measured with Olink proteomics** using whole blood samples of 12 culled minks: animals without clinical signs (NCSc, n=5, #16, 17, 18, 19 and 20), with clinical signs (CSc, n=4, #27, 28, 29 and 30), and found dead (FDc, n=3, #36, 37 and 39 ). The 92 proteins were analyzed using a proximity extension assay (PEA)- based Proseek Multiplex panel, performed by the Olink provider at UMC Utrecht, the Netherlands. The Olink panel is not validated for whole blood samples derived from mink. Rows (protein NPX values transformed to a linear scale) were centred, and unit variance scaling was applied to rows. Both rows and columns were clustered using correlation distance and average linkage.
